# Effect of photobiomodulation treatment in the sublingual, radial artery region, and along the spinal column in individuals with multiple sclerosis: Protocol for a randomized, controlled, double-blind, clinical trial

**DOI:** 10.1097/MD.0000000000010627

**Published:** 2018-05-11

**Authors:** Tamiris da Silva, Fernanda Cordeiro da Silva, Andréa Oliver Gomes, Ariane Oliveira Viana, Marcela Letícia Leal Gonçalves, Maria Fernanda Setúbal Destro Rodrigues, Anna Carolina Ratto Tempestini Horliana, Daniela de Fátima Teixeira da Silva, Maria Cristina Chavantes, Yara Dadalti Fragoso, Luciana Prats Branco, Lara Jansiski Motta, Kristianne Porta Santos Fernandes, Raquel Agnelli Mesquita-Ferrari, Sandra Kalil Bussadori

**Affiliations:** a Nove de Julho University (UNINOVE); b University Metropolitana de Santos (UNIMES) University, Santos, Brazil.

**Keywords:** low-level laser therapy, multiple sclerosis, oxidative stress, photobiomodulation, physical therapy

## Abstract

**Background:**

Multiple sclerosis (MS) is an autoimmune disease, for which the forms of treatment are medication and rehabilitation. However, in vitro and in vivo studies have demonstrated that photobiomodulation can be an effective treatment modality for inflammatory diseases, including MS. Photobiomodulation has a broad range of benefits, such as the avoidance of cell and tissue death, the stimulation of healing and injury repair, reductions in pain, edema and inflammation, cell proliferation, and even apoptosis. The outcomes of photobiomodulation include the regeneration of cells, the stimulation of the growth of Schwann cells, a reduction in spasticity, functional improvements, a reduction in nitric oxide levels, and the upregulation of the cytokine IL10, demonstrating that this therapeutic modality can offer neuroprotection.

**Methods:**

A randomized, controlled, double-blind, clinical trial is proposed. The patients will be divided into 6 groups. Groups 1 and 2 will receive sham and active photobiomodulation in the sublingual region, respectively. Groups 3 and 4 will receive sham and active photobiomodulation along the spinal cord, respectively. Group 5 will receive placebo treatment with photobiomodulation on the skin in the region of the radial artery with a specific bracelet. Group 6 will be treated with photobiomodulation on the skin in the region of the radial artery.

**Discussion:**

Treatment for MS is directed at the immune response and slowing the progression of the disease. This is one of the first clinical trials involving photobiomodulation in the sublingual region and along the spinal cord, which could help establish a promising new form of nonpharmacological treatment for autoimmune diseases. This is one of the first clinical trials with sublingual photobiomodulation and along the spinal cord that could help establish a new form of promising treatment of the disease associated with pharmacological treatment.

## 1 Introduction

Multiple sclerosis (MS) is an demyelinating, neurodegenerative, inflammatory disorder of the central nervous system (CNS) characterized by the selective destruction of the myelin sheath.^[1,2]^ MS has a complex, multifactor etiology that is not fully understood, but it is believed that the formation of demyelinating lesions may be due to autoimmune processes as well as environmental and genetic factors.^[3,4]^

Inflammation of the CNS is an important mechanism that contributes to demyelination and neurodegeneration. Th1, Th17, and B cells are activated in peripheral regions, pass through the blood–brain barrier, and interact with antigen-presenting cells (astrocytes, microglia, macrophages, and dendritic cells), inducing the production of proinflammatory cytokines and oxidative stress.^[5,6]^ B lymphocytes serve as specific antigen-presenting cells for T cells and produce specific antibodies for myelin antigens, making myelin the target of immune cells that mistake it for a foreign antigen.^[6,7]^

Oxidative stress occurs due to the accumulation of free radicals (reactive oxygen and nitrogen species),^[8]^ which leads to inflammation, oligodendrocyte damage, abnormalities in synaptic transmissions, axonal degeneration, and neuronal death, suggesting that oxidative stress is an important factor in neurodegeneration.^[9,10]^ Myelin and oligodendrocyte damage caused by inflammation gives rise to a multitude of symptoms, such as sensory alterations, fatigue, physical and/or mental disability, balance disorders, spasticity, muscle weakness, urinary incontinence, cognitive impairment, neuropathic pain, and visual deficiency.^[1,3]^

This disease occurs in different forms, such as relapsing–remitting MS, which is characterized by sudden-onset short-term or long-term relapses, secondary progressive MS, which has a progressive course that results in severe, irreversible disability, and primary progressive MS, which is a progressive type with no relapses or periods of remission.^[1,11,12]^

Prognosis is unpredictable with regard to the disability that occurs due to the manifestations of the disease, which are normally associated with progressive locomotion impairment.^[12]^ Treatment is directed at the immune response and slowing the progression of the disease, which can be achieved with the use of drugs.^[12,13]^ Moreover, rehabilitation can lead to improvements in walking capacity, cognition, fatigue, depression, quality of life, participation in activities, muscle strength, cardiovascular performance, and balance.^[13,14]^

In vitro and in vivo studies have demonstrated that photobiomodulation is effective for inflammatory diseases, including MS.^[15]^ This therapeutic modality has a broad range of benefits, such as the avoidance of cell and tissue death, the stimulation of healing and injury repair, reductions in pain, edema and inflammation, cell proliferation, and even apoptosis.^[15,16]^ The outcomes of photobiomodulation include the regeneration of cells, the stimulation of the growth of Schwann cells, a reduction in spasticity, functional improvements, a reduction in nitric oxide levels, and the upregulation of the cytokine IL10, demonstrating that this therapeutic modality can offer neuroprotection.^[15,17]^

The aim of the proposed study is to evaluate whether photobiomodulation in the sublingual region and along the spinal cord modulates the expression of IL-10, TNF-α, and nitric oxide in individuals with MS. The main objective of this study is to evaluate if the photobiomodulation along the spinal cord in the sublingual region and irradiation in the radial artery can modulate the expression of IL-10, TNF-α, and nitric oxide.

## 2 Methods/Design

### 2.1 Type of study

A controlled, clinical trial is proposed, which will follow the guidelines for research involving human subjects stipulated in Resolution 466/2012 of the Brazilian National Board of Health and will be submitted for approval from the Human Research Ethics Committee of University Nove de Julho. The participants or their legal guardians will sign statements of informed consent authorizing participation in the study.

### 2.2 Trial registration

Clinical.trials.gov as NCT03360487, first received in 4 December 2017, https://clinicaltrials.gov/ct2/show/NCT03360487.

### 2.3 Sample calculation

**Figure 1:**
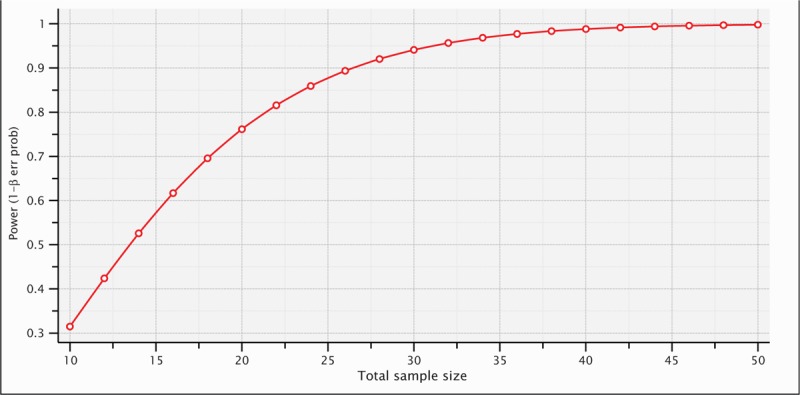
Sample calculation.

The sample size was calculated to ensure a 95% test power. It was determined that with 32 individuals and an effect size of 0.8, the test power would be 0.9566, thereby maintaining the significance level at α = 0.05 (Fig. 1).

*Inclusion criteria*: diagnosis of MS, age between 18 and 60 years, currently undergoing pharmacological treatment, capable of understanding and following verbal instructions and score of <6 on the Expanded Disability Status Scale. No restriction will be imposed regarding gender.

*Exclusion criteria*: other autoimmune disease and/or tumor, relapse of disease activity during treatment, and not undergoing pharmacological treatment. Other autoimmune diseases; neoplasias, heart failure, respiratory failure, renal insufficiency, hepatic insufficiency, acquired immunodeficiency syndrome, and patients with relapses of the disease.

### 2.4 Recruitment and randomization

Patients will be recruited from the Integrated Health Clinic of University. Patients with a diagnosis of MS will be screened for the eligibility criteria through telephone interviews. Selected individuals with a signed statement of informed consent will be randomized. Randomization will be performed in blocks. Groups 1 and 2 will receive sham and active photobiomodulation in the sublingual region, respectively. Groups 3 and 4 will receive sham and active photobiomodulation along the spinal cord, respectively. Group 5 will receive placebo treatment with photobiomodulation on the skin in the region of the radial artery with a specific bracelet. Group 6 will be treated with photobiomodulation on the skin in the region of the radial artery. The participants will not be deprived of any medication for the treatment of the base condition. Randomization will be stratified by clinic using block allocation tables to ensure equal proportions in the distribution of treatments. The participants and evaluator will be blinded to the allocation.

## 3 Evaluations

### 3.1 EDSS

The participants will be evaluated before and after treatment using the Expanded Disability Status Scale administered by a physiotherapist (approximate application time: 15 minutes).

Blood collection for analyzes of inflammation, oxidative stress.

All participants will go through a medical consultation at the UNINOVE clinic, to confirm the diagnosis of MS, and after which it will be collected by a nurse blood samples (10 mL) will also be taken for the determination of IL-10 (anti-inflammatory), TNF-α, and nitric oxide (proinflammatory). The evaluation of these cytokines will be through ELISA and Griess reaction.

### 3.2 Treatment

The participant will be placed on an examining table in a comfortable position. Both the operator and participant will use eye protection.

Transcutaneous irradiation of the spinal cord will be performed on segments corresponding to the nerve roots of the lumbosacral plexus (T12-S5) and cervicothoracic plexus (C5-T1-2). Twenty points will be irradiated for 90 seconds (total treatment time: 1800 seconds). In the group submitted to sublingual irradiation, disposable plastic wrap will cover the application pen for the purposes of hygiene, (total treatment time: 360 seconds). In the group submitted photobiomodulation on the skin in the region of the radial (total treatment time: 360 seconds).

The treatment will be performed twice a week totaling 24 consecutive weeks and after 3 months of treatment the patients will be submitted to reassessment of all complementary tests that have been requested, cytokines, nitric oxide, and EDSS.

### 3.3 Photobiomodulation protocol

With regard to the photobiomodulation protocols, the articles of interest were identified through a bibliographic survey of titles and abstracts. The selected articles were analyzed and used for the establishment of the treatment protocol (Table 1).

**Table 1:**
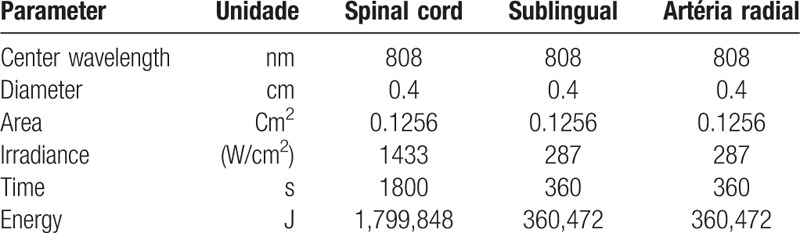
Laser parameters.

### 3.4 Statistical analysis

The data will be tabulated and treated using the SPSS 20.0 for Windows. Descriptive statistics will be performed. The chi-square test and Fisher exact test will be used to test associations with the categorical variables. The Student *t* test will be used and Pearson's correlation coefficients will be calculated for the analysis of correlations among the continuous variables. The level of significance will be set at 95% (*P* < .05).

## 4 Discussion

Treatment for MS is directed at the immune response and slowing the progression of the disease. This is one of the first clinical trials involving photobiomodulation in the sublingual region and along the spinal cord, which could help establish a promising new form of nonpharmacological treatment for autoimmune diseases.

## 5 Declarations

### 5.1 Ethics committee

The Ethics Committee of the University of Nove de Julho (UNINOVE) approved this project with number 2.423.755, in accordance with the guidelines of the National Ethics Committee (CONEP). Any modifications to the protocol that may have an impact on the conduct of the study will be reported to the UNINOVE Ethics Committee.

Informed consent/consent (which was approved by the UNINOVE Ethics Committee) will be signed by the volunteers on the day of the interview.

The biological material (blood) will be discarded after the study.

### 5.2 Data collection methods

The authors were previously trained to collect data and a trained professional for blood collection. All authors are qualified in laser therapy.

All data will be entered electronically. The participants’ files will be stored in numerical order in a safe place and accessible only to the authors of this study.

### 5.3 Discontinuing intervention

If volunteers become ill or do not adapt to therapy, it will not be possible to continue laser therapy.

### 5.4 Availability of data and materials

The datasets generated and analyzed during the present study are available from the corresponding author at reasonable request. After the analysis of the data, volunteers will be invited to a meeting and the results will be shared and they will become public.

## Acknowledgments

To the University Nove de Julho (UNINOVE) for the availability of laboratories and volunteers.

## Author contributions

Conceive and design the study: TS, SKB, RAMF, MFSDR; will perform the experiment: TS, FCS, YDF, MLLG, MFSDR, SKB; will analyze the data: TS, ACRTH, DFTS, MCX, AOG; will perform the statistical analysis: TS, ACRTH, DFTS, AOV, LPB, SKB; write the paper: TS, AOG, FCS, RAMF, MLLG, SKB.

**Conceptualization:** Tamiris da Silva, Ariane Oliveira Viana, Maria Fernanda Setúbal Destro Rodrigues, Maria Cristina Chavantes, Sandra Kalil Bussadori.

**Data curation:** Anna Carolina Ratto Tempestini Horliana.

**Formal analysis:** Maria Fernanda Setúbal Destro Rodrigues, Luciana Prats Branco.

**Investigation:** Tamiris da Silva, Ariane Oliveira Viana.

**Methodology:** Tamiris da Silva, Andréa Oliver Gomes, Ariane Oliveira Viana, Maria Cristina Chavantes.

**Project administration:** Tamiris da Silva, Maria Cristina Chavantes, Sandra Kalil Bussadori.

**Resources:** Kritianne Porta Santos Fernandes.

**Software:** Marcela Leticia Leal Gonçalves, Anna Carolina Ratto Tempestini Horliana.

**Supervision:** Tamiris da Silva, Fernanda Cordeiro da Silva, Sandra Kalil Bussadori.

**Validation:** Tamiris da Silva, Daniela de Fátima Teixeira da Silva, Yara Dadalti Fragoso, Lara Jansiski Motta.

**Visualization:** Fernanda Cordeiro da Silva, Andréa Oliver Gomes, Ariane Oliveira Viana, Marcela Leticia Leal Gonçalves, Daniela de Fátima Teixeira da Silva, Lara Jansiski Motta, Kritianne Porta Santos Fernandes.

**Writing – original draft:** Tamiris da Silva, Fernanda Cordeiro da Silva, Andréa Oliver Gomes, Raquel Agnelli Mesquita-Ferrari, Sandra Kalil Bussadori.

**Writing – review and editing:** Andréa Oliver Gomes, Yara Dadalti Fragoso, Kritianne Porta Santos Fernandes, Raquel Agnelli Mesquita-Ferrari, Sandra Kalil Bussadori.
